# P-1045. Death by CLABSI?

**DOI:** 10.1093/ofid/ofaf695.1240

**Published:** 2026-01-11

**Authors:** Natalie Ross, Holly Meacham, Kristin Varzavand, Elizabeth Krigbaum, Olivia Wulf, Philip M Polgreen, Karen Brust

**Affiliations:** University of Iowa Hospitals and Clinics, Iowa City, Iowa; University of Iowa Hospitals and Clinics, Iowa City, Iowa; University of Iowa Hospitals and Clinics, Iowa City, Iowa; University of Iowa Hospitals and Clinics, Iowa City, Iowa; University of Iowa Hospitals and Clinics, Iowa City, Iowa; University of Iowa Carver College of Medicine, Iowa City, IA; University of Iowa Hospitals & Clinics, Iowa City, Iowa

## Abstract

**Background:**

Central line-associated bloodstream infections (CLABSIs) are hospital-acquired infections tied to quality metrics and penalties, but their validity near the end of life is unclear. We hypothesized that critically ill patients experience gut translocation of mucosal barrier injury (MBI) organisms, leading to bloodstream infections misclassified as CLABSIs. We evaluated the microbiology near death to assess the impact of gut translocation on reporting.

**Figure 1:**
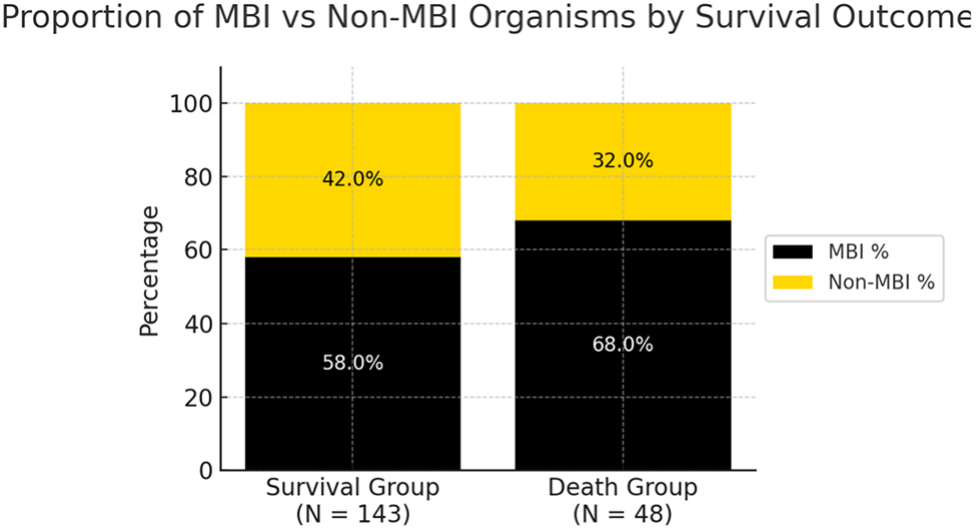

**Figure 2:**
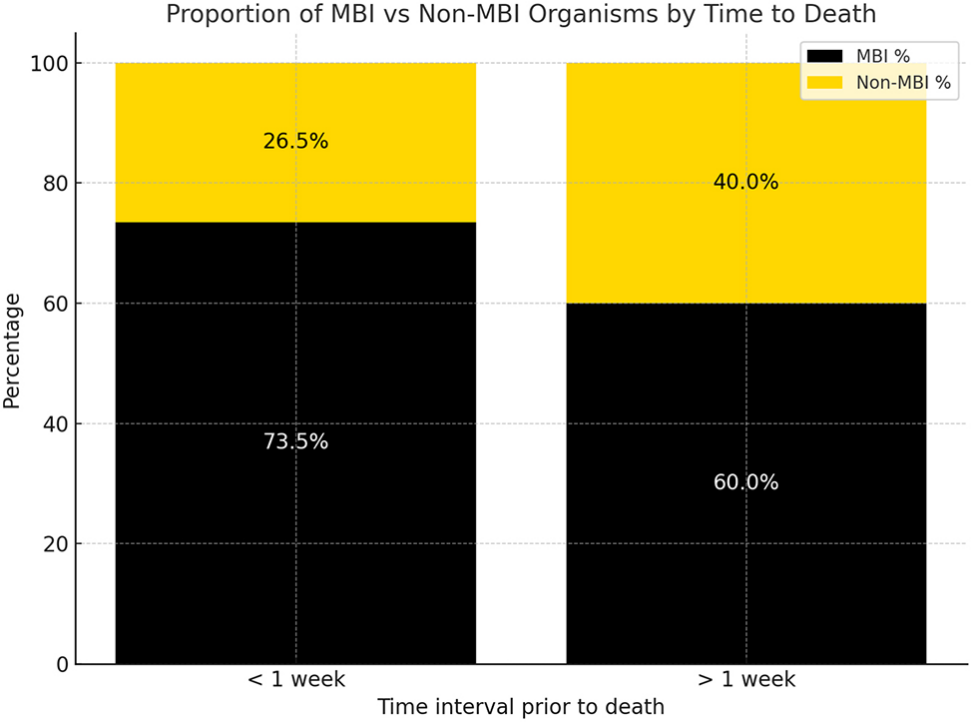

**Methods:**

A retrospective review of 191 adult inpatients with mucosal barrier injury (MBI) and non-MBI CLABSIs at University of Iowa Hospitals and Clinics (2021–2025) was conducted using National Healthcare Safety Network (NHSN) surveillance definitions. Patients were divided into survival and death groups, and blood culture data were analyzed relative to the time of death. Organisms were classified as MBI or non-MBI per NHSN criteria.

**Table 1:**
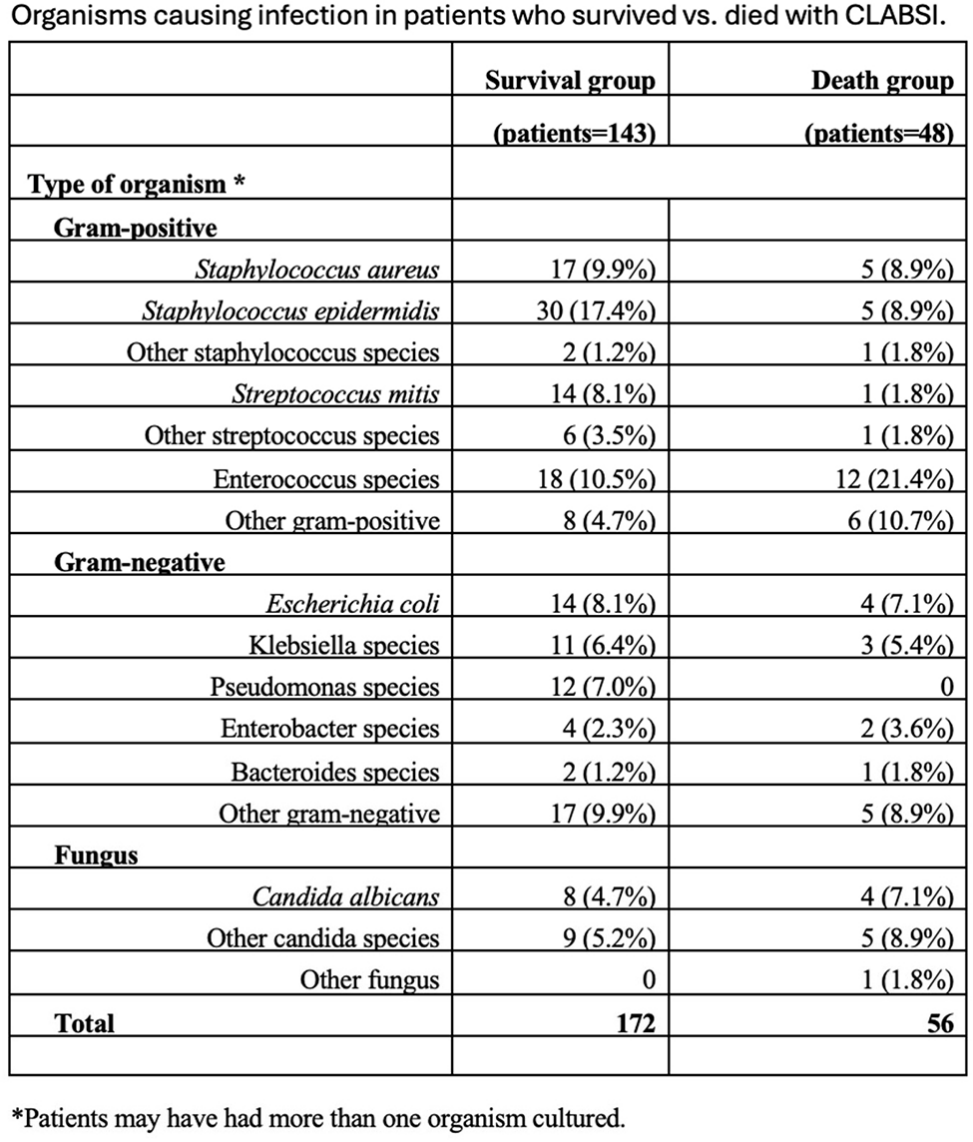

**Results:**

Of 191 patients, 48 (25%) died during admission, with 11 patients dying before the blood culture had resulted. Relative to the survival group, MBI organisms accounted for a greater proportion of bloodstream infections (68.0% vs. 58.0%, Figure 1). In the death group, CLABSIs occurring within 1 week of death were more often caused by MBI organisms (73.5%, Figure 2). Organism-specific analysis is shown in Table 1.

**Conclusion:**

Data demonstrates that MBI organisms are more frequently isolated as patients near death, suggesting that CLABSIs may result from gut translocation rather than true catheter infections. These findings warrant review of CLABSI surveillance definitions near the end of life to improve reporting accuracy.

**Disclosures:**

Philip M. Polgreen, MD, Eli Lily: Advisor/Consultant|Pfizer: Grant/Research Support

